# Impact of a 24 h feed withdrawal on active nutrient transport, intestinal morphology, and gene expression in the equine small and large intestine

**DOI:** 10.1093/tas/txad003

**Published:** 2023-01-28

**Authors:** Blaire E Aldridge-Dean, Timothy B Lescun, John Scott Radcliffe

**Affiliations:** BSM Partners, Bentonville, AR 72712, USA; Department of Veterinary Clinical Sciences, Purdue University, West Lafayette, IN 47907, USA; Department of Animal and Food Science, University of Kentucky, Lexington, KY 40546, USA

**Keywords:** absorption, equine, feed withdrawal, gene expression, intestine, nutrient

## Abstract

Horses are often subjected to short-term feed withdrawal (FW) pre- or post-surgery to reduce anesthetic complications. However, removing nutrients from the intestinal lumen may negatively impact intestinal health. Thirteen horses were used to determine the effects of a 24 h FW on gut barrier function, active nutrient transport, transporter gene expression, and intestinal morphology. Following 0 or 24 h FW (0FW or 24FW, respectively), horses were euthanized via overdose of sodium pentobarbital and sodium phenytoin, and segments of proximal jejunum (PJ), mid jejunum (MJ), ileum (Il), and right ventral colon (RVC) were harvested for histology (PJ and Il), gene expression, and active nutrient transport analysis. Active transport measurements were determined using modified Ussing chambers following the addition of glucose, phosphorus, glutamine, and Gly-Sar. Carbachol-induced chloride (Cl) ion secretion was measured to examine the diarrhetic response. Messenger RNA expression of the intestinal Na-dependent glucose cotransporter (SGLT1), fructose transporter (GLUT5), di- and tri-peptide transporter (PepT1), and neutral AA/glutamine transporter (ASCT2) were determined using RT-PCR. The GLM procedure of SAS was used to determine the effects of FW and responses among various intestinal sections. The horse served as the experimental unit. Villus heights (*P *< 0.002) and crypt depths (*P *< 0.02) in the Il were larger than in the PJ, though no differences were observed between 0FW and 24FW horses. Active glutamine absorption increased 82% in the PJ of 24FW horses compared to 0FW horses (*P *< 0.02). The mRNA expression of SGLT1 decreased (*P *< 0.05), moving aborally in the gastrointestinal tract. Horses subjected to 24FW had 82% less GLUT5 (*P *< 0.05) and 61% less PepT1 mRNA expression in the PJ, compared to 0FW horses. Interestingly, ASCT2 mRNA expression increased 164% from PJ to RVC (*P *= 0.05). However, a 36% decrease in ASCT2 mRNA expression was observed overall for 24FW horses. These data indicate that SGLT1, GLUT5, PepT1, and ASCT2 are expressed throughout the small intestine and RVC of the horse at varying concentrations and that they can be differentially regulated by a 24 h FW. Data from this experiment also indicate that a 24 h FW results in up regulation of active glutamine absorption, presumably in an effort to supply glutamine as an energy substrate for enterocytes.

## INTRODUCTION

Horses are routinely subjected to short-term feed withdrawal (FW) for medical or management reasons. The removal of nutrients from the gastrointestinal (GI) lumen may negatively impact intestinal function, leading to increased disease susceptibility. Knowledge of how FW alters morphological and cellular changes in the intestinal tract of horses is limited. In addition, the capacity of the equine GI tract to absorb nutrients has been poorly examined, with few exceptions (Dyer et al., 2002; Woodward et al., 2008, 2010). The role of the GI tract is multifaceted, having key functions in the regulation of nutrient absorption, immune surveillance capability (Failla, 2003), and protection against pathogens. Molecular techniques can be used to expand the understanding of intestinal function in the horse. Specifically, responses in nutrient transport capacity and nutrient transporter gene expression to various stimuli such as 24 h FW.

During times of stress, it is unclear how nutrient needs are altered within the GI tract. Glutamine, a nonessential amino acid, apparently becomes a major energy substrate during catabolic stress in Caco-2 cells (Sakiyama et al., 2009). Depletion of glutamine has also been reported to increase the permeability of the intestine (van der Hulst et al., 1993). In broilers, Thompson and Applegate (2006) reported changes in intestinal morphology and a linear decrease in mucus content as feed withdrawal times were increased from 0 to 24 h, stressing the need for a better understanding of the impact of FW on morphology, nutrient uptake and nutrient transporter gene expression. Therefore, our goal was to determine the effects of FW in various regions of the small and large intestine on GI morphology, the active transport capacity of core nutrients, as well as intestinal transporter mRNA expression of the Na-dependent Glc transporter 1 (SGLT1), fructose transporter (GLUT5), di- and tri-peptide transporter (PepT1) as well as ASCT2. It was hypothesized that a 24 h FW would alter intestinal function in the horse and that the response would differ among segments of the intestinal tract in the horse.

## MATERIALS AND METHODS

### Collection of Tissue

Thirteen Quarter horse or Quarter horse cross geldings (5 to 15 yr of age) were used to determine the effects of a 24 h FW on GI morphology, transport capacity of core nutrients, as well as various intestinal transporter mRNA. Following an 8-wk period of ad libitum grass hay consumption, six horses were provided hay, and seven horses were denied access to hay for 24 h prior to euthanasia. All horses were consuming hay from the same bales for the 8-wk time period. They were individually housed in stalls and provided hay twice daily. No feed differences in feed intake were observed, but feed intake was not specially measured. All animal procedures were approved by the Purdue Animal Care and Use Committee. Horses were sedated with xylazine hydrochloride (1 mg/kg BW, IV) and then euthanized with an overdose of sodium pentobarbital (39 g, IV) and sodium phenytoin (5 g, IV). Four sections of the equine small and large intestine were collected: 1) proximal jejunum (PJ: 13.7 m proximal to the ileo-cecal junction), 2) mid jejunum (MJ: 9.1 m proximal to the ileo-cecal junction), 3) ileum (Il: 5 cm proximal to the ileo-cecal junction), and 4) right ventral colon (RVC: 30 cm distal to the cecocolic junction). Intestinal segments taken for Ussing chambers were immediately placed in ice-cold aerated Ringers buffer (mM: CaCl_2_, 1.2; MgCl_2_, 1.2; Na_2_HPO_4_, 2.4; NaH_2_PO_4_, 0.4; NaHCO_3_, 25; KCl, 5; NaCl, 115; 7.4 pH) until mounted into chambers. The mucosa was rinsed with ice-cold sterile saline (0.90% NaCl), scraped with a glass slide, and flash frozen in 1 mL of Tri-Reagent for RNA isolation. A 5 cm long segment of the PJ and Il were removed and placed into NoTox fixative (Earth Safe Industries, Inc., Belle Mead, NJ) for histological analysis.

### Histology

Duplicate sets of 5 cm sections of proximal jejunal and ileal tissue were rinsed and placed in NoTox fixative on a shaker for 48 h. Samples were then dehydrated by a graded alcohol series of 70, 80, 95, and 100% ethanol, were cleared with a Sub-X clearing agent (Surgipath Medical Industries, Richmond, IL), and subsequently embedded in paraffin. Tissues were sectioned at 5 µm and floated onto slides which were then stained with hematoxylin and eosin (Sigma-Aldrich, St. Louis, MO). The heights of six villi and depth of six crypts were determined in duplicates per intestinal section.

### Ussing Chambers

The outer serosal layer was removed, tissue was cut along the mesenteric border, and each intestinal section was mounted in duplicate on Ussing chamber inserts, allowing 1 cm^2^ of tissue exposure. Tissues were then mounted into modified Ussing Chambers (VCC MC8; Physiologic Instruments, San Diego, CA) and bathed in 8 mL of oxygenated Ringers buffer, pH 7.4 (95% O_2_/5% CO_2_). A recirculating water bath (VWR, Batavia, IL) held the buffer temperature of the Ussing chambers at 38 °C. Chambers were connected to dual channel voltage/current clamps, and the voltage was clamped at 0 mV by an external current. Following a 10 to 15 min equilibration period, basal transepithelial short-circuit current (Isc, µA/cm^2^) and resistance (TER, ohm/cm^2^) were measured using Acquire and Analyze software (Physiologic Instruments, San Diego, CA). Total ion flux is indicative of active transport, which was measured based on the change in short circuit current (Isc), following the addition of 10 mM of inorganic phosphate (Na_2_HPO_4_), glutamine, or Glycyl-Sarcosine (unhydrolyzable di-peptide) to the mucosal buffer. Nutrient challenges were sequential and osmotically balanced in the serosal buffer with the equivalent concentrations of mannitol. Following a nutrient challenge, researchers waited (~10 min) for a new steady-state short-circuit current to be established prior to initiating the next challenge. The addition of 10 mM carbachol (carbamoylcholine chloride) to the serosal buffer induces chloride ion secretion and was utilized to verify tissue viability, in addition to being indicative of the diarrhetic response in the intestine.

### Real-Time PCR

Total RNA was isolated by TRIzol via the manufacturer’s instructions and cleaned with the RNeasy Mini Kit and RNase-Free DNase Set (Qiagen GmbH, Germany). A NanoDrop (ThermoScientific, Wilmington, DE) was used to quantify RNA, which was then stored at −80 °C, until first-strand complementary DNA (cDNA) was reverse transcribed from total RNA (1 µg) as previously described (Fleet and Wood, 1999).

Primers utilized to determine mRNA abundance were designed based on the DNA sequence deposited at www.ensembl.org (Fernandez-Suarez and Schuster, 2010). Primers were designed by the software Primer 3 (Untergrasser et al., 2012) and were designed to span introns and exons. An iCycler real-time machine (Biorad, CA) was utilized for all real-time reactions using IQ SYBR Green Supermix kit (Biorad, CA). Cycle threshold (Ct) values were used using the method of Livak and Smittgen (2001) to determine the abundance of the sodium-dependent glucose transporter SGLT-1 (SLC5A1), fructose transporter GLUT5 (SLC2A5), di- and tri-peptide transporter PepT1 (SLC15A1), sodium-dependent neutral amino acid/glutamine transport system ASCT2 (SLC1A5), as well as the glyceraldehyde 3-phosphate dehydrogenase (GAPDH) housekeeping gene (Livak and Smittgen, 2001). Sample Ct values were normalized to the expression of GAPDH, and 2^-ΔCt^ values were expressed as arbitrary units (Cui et al., 2009). In order to validate primer sets and ensure a single amplicon, melt curve analysis and an agarose gel were used throughout RT-PCR analysis to confirm single product identities as previously described (Healy et al., 2003). Primers utilized were SGLT-1: forward: 5ʹ-TGTCGGGCTGTGGGCTAT-3ʹ, reverse: 5ʹ-TTCGTCCTGCGAGGAAGAAG-3ʹ, annealing temperature (Ta) = 53.1 °C; GLUT5: forward: 5ʹ-CCGCCATCTTAATGGGAAC-3ʹ, reverse: 5ʹ-GACAACGTTGGAAGACAGACC-3ʹ, annealing temperature (Ta) = 51.0 °C; PepT1: forward: 5ʹ-GGATGCTGTGGTGTATCCTCT-3ʹ, reverse: 5ʹ-CATCCCAACCGTCATCTTCT-3ʹ, annealing temperature (Ta) = 53.0 °C; ASCT2: forward: 5ʹ-GTGTGTGGAGGAGAAGAATGG-3ʹ, reverse: 5ʹ-ATGAGGGCCAGAGTGAGGA-3ʹ, annealing temperature (Ta) = 58.1 °C; GAPDH: forward: 5ʹ-TCCCTGCTTCTACTGGTGCT-3ʹ, reverse: 5ʹ-GAGACAACCTGGTCCTCAGT-3ʹ, annealing temperature (Ta) = 55.9 °C.

### Statistical Analysis

The experiment was a completely randomized design, whereby the main effects of the intestinal section, treatment (FW), and section × treatment were analyzed using the GLM procedure of SAS (SAS Inst., Inc, Cary, NC). The experimental unit was the horse intestine section. Differences were considered significant at *P* < 0.05 and trends at *P* < 0.10. All data are reported as LSmeans ± SE.

## RESULTS

### Histology

**Figure 1.**
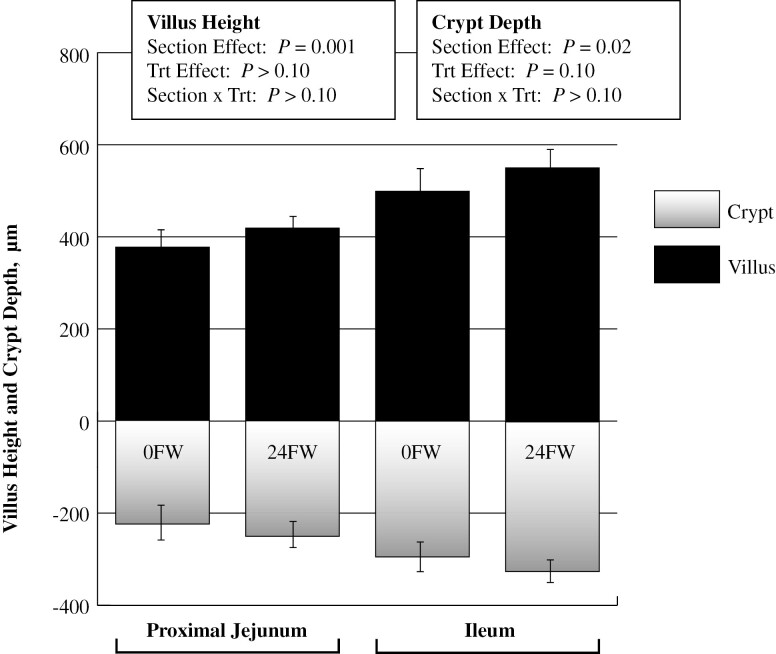
The effects of a 24 h feed withdrawal (24FW) versus 0 h FW (0FW) on villus height and crypt depth. Villi were longer (*P* < 0.01) and crypts were deeper (*P* < 0.05) in the ileum compared to the proximal jejunum. No effects (*P* > 0.10) of FW on villus heights or crypt depths were observed. Values are reported as LSmeans ± SE, *n* = 7 for 24 h FW; *n* = 6 for 0 h FW.

Villus heights (*P* < 0.001) and crypt depths (*P* < 0.02) were 34 and 26% larger in the Il compared to the PJ, respectively. No differences in villus height or crypt depth were observed between 0FW and 24FW horses (Figure 1).

### Ussing Chambers

**Figure 2.**
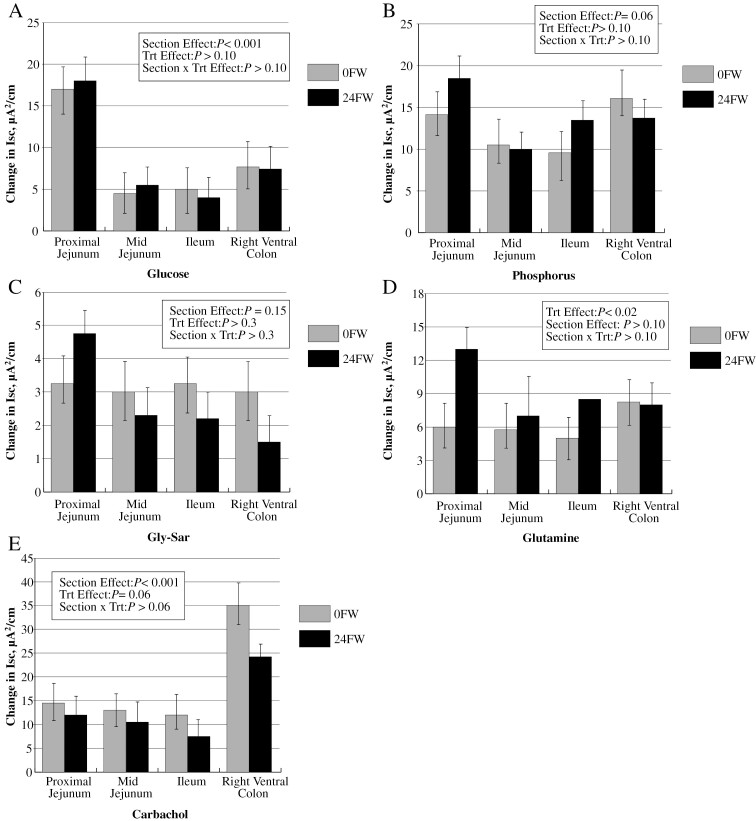
Effect of a 24 h feed withdrawal (24FW) versus 0 h FW (0FW) on equine intestinal active transport measurements, as indicated by the change in short circuit current (ΔIsc, µA/cm^2^), following the addition of 10 mM (A) glucose, (B) inorganic phosphate (Na_2_HPO_4_), (C) the unhydrolyzeable di-peptide glycyl-sarcosine (Gly-Sar), and (D) glutamine to the luminal buffer. Ten mM carbachol-induced chloride ion secretion was added to the serosal buffer (E), and is indicative of the diarrhetic response. All challenges were osmotically balanced with 10 mM mannitol in the opposing chamber (serosal side). Values are reported as LSmeans ± SE, *n* = 7 for 24FW; *n* = 6 for 0FW.

The change in short circuit current (ΔIsc, μA/cm^2^), indicative of active transport, was determined for intestinal tissue mounted in modified Ussing chambers following the addition of glucose, phosphorus, glutamine, and glycl-sarcosine (Gly-Sar). Carbachol-induced Cl^-^ secretion was also determined. Feed withdrawal had no effect (*P* > 0.10) on the active absorption of glucose, phosphorus, or Gly-Sar (Figure 2). Active glucose transport was ~267% greater (*P* < 0.001) in the PJ compared to the Il and MJ (Figure 2A). Active phosphate transport was 48% greater in the PJ (*P* < 0.06) and 36% greater in the RVC (*P* < 0.06), compared to the MJ and Il (Figure 2B). Differences in active Gly-Sar transport were not observed, though they did approach a trend (*P* = 0.14) for being greater in the PJ with similar transport capacities in all the other sections (Figure 2C). Feed withdrawal (24 h) stimulated a 116% increase (*P* < 0.02) in active glutamine transport in the PJ (Figure 2D) compared to 0FW horses. There was a trend (*P* < 0.07) for carbachol-stimulated chloride ion secretion to be greater for 0 h FW compared to 24 h FW horses. More (*P* < 0.001) chloride ion secretion was observed in the RVC compared to all other sections (Figure 2E).

### Gene Expression

**Figure 3.**
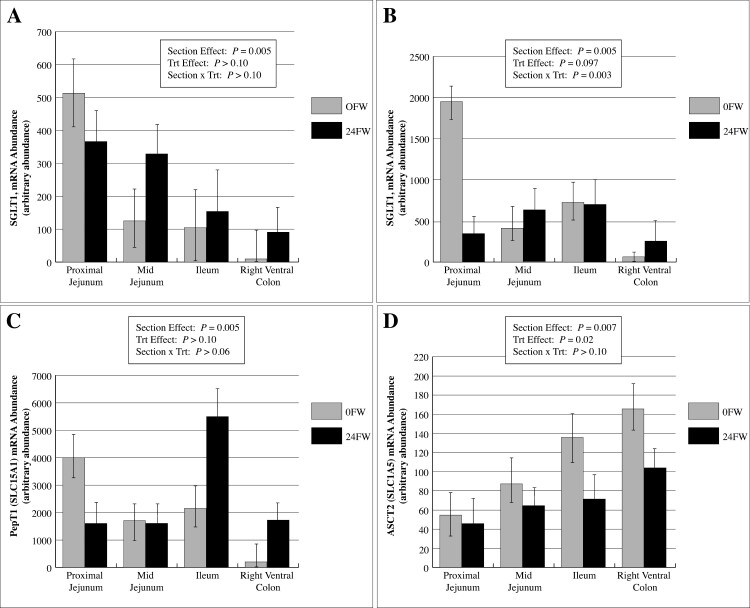
Effect of a 24 h feed withdrawal (24FW) versus 0 h FW (0FW) on equine intestinal core nutrient transporter mRNA expression, normalized to *GAPDH*, of the (A) sodium-dependent glucose cotransporter SGLT1 (*SLC5A1*), (B) fructose transporter GLUT5 (*SLC2A5*), (C) the di- and tri-peptide transporter PepT1 (*SLC15A1*), and (D) neutral amino acid (and glutamine) transporter ASCT2 (*SLC1A5*). Values are reported as LSmeans ± SE, *n* = 7 for 24FW; *n* = 6 for 0FW.

There was no difference (*P* > 0.10) in SGLT1 mRNA expression between 0FW and 24FW horses (Figure 3). However, SGLT1 expression decreased (*P* < 0.01) from the proximal to distal sections (PJ > MJ > Il > RVC) of the GI tract (Figure 3A). Overall, there was approximately 221% more SGLT1 mRNA, relative to GAPDH, in the PJ than in the Il, and 71% more SGLT1 mRNA in the MJ compared to the Il (Figure 3A). A section-by-treatment interaction was observed in GLUT5 gene expression (*P* = 0.02) for horses subjected to 24 h FW (Figure 3B). These horses had an 82% decrease in GLUT5 mRNA gene expression in the PJ compared to 0 h FW horses. Additionally, there was a trend (*P* = 0.06) for a section by treatment interaction on mRNA abundance of PepT1 with a 61% decrease in mRNA abundance in the proximal jejunum, which was reversed in the Il (Figure 3D). No interaction was observed for sodium-dependent neutral amino acid transporter ASCT2 (SLC1A5) gene expression, though both a section effect (*P* = 0.006) and treatment effect (*P* = 0.02) were observed (Figure 3E). Interestingly, a 164% increase in ASCT2 gene expression was observed in the RVC compared to the PJ (Figure 3E). A 36% overall drop in ASCT2 gene expression was exhibited in 24FW horses.

## DISCUSSION

To improve nutrition and feeding management practices in horses, knowledge regarding how and where nutrients are absorbed, utilized, and regulated under varying physiologic stressors (feed withdrawal, disease, exercise, reproductive status, etc.) is needed. The intestinal epithelium provides a physiological barrier against pathogens, and its health and integrity are paramount in health status (Burkholder et al., 2008). The role of FW on prime nutrient absorption locations, respective physiological function (active transport capacity), coupled with understanding the regulation of transporter gene expression, helps to explain intestinal adaption and regulation in response to decreased nutrient load. While each nutrient maintains a specific function and role, representation of core nutrients was sought for sugars, minerals, amino acids, di-peptides, and chloride ion secretion (diarrhetic response). To our knowledge, this is the first time that the active transport capacity of select nutrients due to FW has been examined in the horse, as well as gene expression of PepT1 and ASCT2. In addition, previously unidentified absorption capabilities for amino acids and peptides were shown to exist in the hindgut of the horse.

The active transport capacity of glucose, an energy substrate in eukaryotic cells (Dyer et al., 2003), is greatest in the PJ of the horse but is not affected by FW. Dyer et al. (2009) demonstrated the capacity for SGLT1 to be adaptable at both the protein level and mRNA expression if horses were fed diets that were mostly carbohydrates rather than hay. To our knowledge, regulation responses to short-term FW have not been previously characterized in the horse. Gene expression of SGLT1 tended to be upregulated in the MJ of feed-restricted horses, which agreed with responses of SGLT1 in feed-deprived birds (Gal-Garber et al., 2000). However, no functional change was observed at the physiological level of glucose absorptive capacity. Dyer et al. (2002) also observed the greatest glucose absorption capacity from brush border membrane vesicles in the PJ of the horse. As 0FW horses were maintained on grass hay, it is unclear how the removal of concentrated feeds might have altered these results. Dyer et al. (2002) observed decreasing SGLT1 mRNA expression from the duodenum > jejunum > ileum, which is in agreement with our findings. Quantification of SGLT1 mRNA expression in the right ventral colon of the horse is a novel finding of our study.

As fructose is not actively transported through GLUT5, transport measurements were not available to evaluate the section by treatment differences observed in GLUT5 gene expression. Merediz et al. (2004) observed a decreased GLUT5 mRNA abundance throughout the small intestine (jejunum > ileum) in the horse, which correlated with protein expression. The GLUT5 is regulated by its own substrate (fructose) (Douard and Ferraris, 2008), which supports the 5.6-fold downregulation of GLUT5 gene expression in PJ of 24FW horses. GLUT5 is regulated transcriptionally (Jiang and Ferraris, 2001) and is stimulated by the presence of fructose (Shu et al., 1998); therefore, it is not surprising that the intestinal section (PJ) first exposed to the removal of nutrients would be the section first affected.

A 24 h FW did not affect intestinal morphology in either the PJ or Il, indicating the gut integrity is not yet altered. Villus height and crypt depth were higher in the Il than PJ, however. At a 24 h time point, it seems that gut integrity is not yet altered. Of particular interest is the fact that enterocytes may become glutamine deficient within 24 h of feed removal, which indicates an impending deterioration of gut function. Glutamine is used as an energy substrate by enterocytes and plays a major role in proliferation and repair (Clark et al., 2003). When stimulated by TNF-α, glutamine depletion induces *E. coli* translocation across Caco-2 cells (Clark et al., 2003), supporting its protective role in the gut. In broilers, the mucin layer in the Il has been reported to decrease as the duration of FW increases, thereby influencing its ability to protect the epithelium from various pathogens and altering nutrient absorption (Thompson et al., 2004). A study by Burkholder et al. (2008) found a 24 h FW increased salmonella attachment indicating the role FW may play in increasing pathogen attachment rates. Data from the current experiment indicate that a 24FW results in upregulation of active glutamine absorption throughout the small intestine, most prominently in the proximal small intestine. Feed withdrawal (24 h) increased active glutamine transport in the small intestine (PJ, MJ, and Il) by 120, 60, and 40%, respectively, though no change was observed between 0FW and 24FW horses in the RVC. Howard et al. (2004) observed an increase in the apical membrane transporter ASCT2 in times of luminal starvation. The ASCT2 (and PepT1) was upregulated in the distal intestine, which was observed in the current study as well. Interestingly, FW reduced ASCT2 gene expression by 35% in 24FW horses, indicating that a 24 h FW reduces the gut energy substrate glutamine enough for enterocytes to physiologically increase its transport capacity, but not by upregulation of ASCT2 gene expression. Glutamine itself promotes the upregulation of ASCT2 gene expression in cell culture work (Bungard and McGivan, 2004), so it is plausible the lack of glutamine causes a downregulation in expression, which may, in turn, upregulate apical membrane transporters. In general, glutamine has similar absorptive capacities in both the small and large intestines, with ASCT2 gene expression increased by 162% in the RVC compared to the PJ, indicating the potentially significant role of the hindgut for glutamine absorption.

The unhydrolyzable peptide Gly-Sar has been shown to be transported through the PepT1 transporter (Irie et al., 2005) and is an indicator of di-peptide transport. An increase in PepT1 mRNA abundance was observed in feed-restricted chickens (Gilbert et al., 2008), and an increased PepT1 protein expression was reported in fasted rats (Ogihara et al., 1999; Thamotharan et al., 1999; Ihara et al., 2000). While Gly-Sar transport was detected in both the small and large intestines, feed restriction tended to increase Gly-Sar transport in the PJ of horses. Gene expression of PepT1 was 147% greater in the Il of feed-restricted horses, but was reversed in the PJ. A larger sample size might confirm these novel findings. While PepT1 gene expression and di-peptide transport in the RVC is a novel finding in the horse, Woodward et al. (2010) recently reported mRNA transcript abundances of a variety of amino acid transport systems in the hindgut (left dorsal and left ventral colon) of the horse. These previously uncharacterized findings signify the importance of the hindgut in amino acid and peptide transport.

Schryver et al. (1974) and Hintz (1975) reported evidence of phosphorus absorption in the hindgut of the horse, but little to no work has since been reported. While a trend, our observations that phosphate absorptive capacity was greatest in the PJ and RVC, further emphasizes the importance of the RVC in nutrient absorption. Implications from the results emphasize the potential importance of phosphorus, peptide, and amino acid absorption in the horse, and highlight the role of the hindgut in nutrient absorption. Requirements for protein (from peptides and amino acids) and phosphorus may need to be reexamined to include hindgut nutrient utilization, particularly for additional absorptive benefits of mineral, protein, and amino acid utilization within fibrous feedstuffs. For example, recommendations on phosphorus requirements may need to be altered to account for the potential microbial fermentative release of phytate-bound phosphorus (van Doorn et al., 2004) and the capacity of phosphorus transport in the hindgut of the horse?

This novel research is the first to quantitatively describe the impact of a 24 h FW on intestinal core nutrient transporter gene regulation and function in the horse. Gene expression data indicate that SGLT1, GLUT5, PepT1, and ASCT2 are expressed throughout the small intestine and RVC of the horse at varying concentrations and that the mRNA expression of these transporters was differentially regulated by a 24 h FW. Data from this experiment also indicate that a 24 h FW results in an upregulation of active glutamine absorption in an effort to increase the availability of this major energy substrate for enterocytes.
